# A chemical family-based strategy for uncovering hidden bioactive molecules and multicomponent interactions in herbal medicines

**DOI:** 10.1038/srep23840

**Published:** 2016-03-30

**Authors:** Hui-Peng Song, Si-Qi Wu, Haiping Hao, Jun Chen, Jun Lu, Xiaojun Xu, Ping Li, Hua Yang

**Affiliations:** 1 State Key Laboratory of Natural Medicines (China Pharmaceutical University), No. 24, Tongjia Lane, Jiangsu, Nanjing 210009, China

## Abstract

Two concepts involving natural products were proposed and demonstrated in this paper. (1) Natural product libraries (e.g. herbal extract) are not perfect for bioactivity screening because of the vast complexity of compound compositions, and thus a library reconstruction procedure is necessary before screening. (2) The traditional mode of “screening single compound” could be improved to “screening single compound, drug combination and multicomponent interaction” due to the fact that herbal medicines work by integrative effects of multi-components rather than single effective constituents. Based on the two concepts, we established a novel strategy aiming to make screening easier and deeper. Using thrombin as the model enzyme, we firstly uncovered the minor lead compounds, potential drug combinations and multicomponent interactions in an herbal medicine of Dan-Qi pair, showing a significant advantage over previous methods. This strategy was expected to be a new and promising mode for investigation of herbal medicines.

Natural product libraries (NPLs) have historically been an invaluable source of drug candidates^1^,^2^. Almost half of the small-molecule drugs in use today are directly or indirectly derived from NPLs^3^, and it is believed that NPLs will continuously play a highly important role in drug discovery. However, the fact is that the contribution of NPLs to drug discovery has declined in recent decades^4^,^5^, leading to further reduction of technology investment by many large pharmaceutical companies^6^. The reason for this involves two aspects. First, some relatively complex NPLs are not so “screen friendly”, in which the structures of compounds remain unclear and their contents range from trace level to milligram level, resulting in technical obstacles such as the poor compatibility with high-throughput screening^2^,^7^,^8^. Second, although numerous methods such as molecular bio-chromatography and computer-aided drug design have been established for activity screening, one non-ignorable fact is that each technology has its weaknesses and inapplicable compound libraries^9^,^10^. Because of the inappropriate application, some highly potential lead compounds are inadvertently missed^11^. Therefore, making the NPLs more compatible with modern screening methods and improving the applicability of technologies are the keys to accelerate drug discovery.

Different from other natural products, traditional herbal medicines have accumulated long-time and large-scale clinical experience in some ancient countries, and thus the therapeutic efficacy, tolerance and safety are relatively better known^12^,^13^. New and creative drug screening strategies inspired by herbal medicines are receiving increasing attention worldwide^14^,^15^,^16^. As a result, numerous studies have reported the successful establishment of efficient methodologies for screening lead compounds in recent years^17^,^18^, by which considerable bioactive small molecules were discovered^19^,^20^. Nevertheless, in ancient medical systems, the therapeutic efficacies of herbs are achieved by combinatorial components rather than single compound^21^,^22^,^23^. For instance, drug compatibility (Pei-Wu in Chinese), which refers the relationships between drugs such as mutual reinforcement, mutual inhibition and mutual restraint, is used as a predominant remedy in traditional Chinese medicines, one of the ancient medical systems with thousand-year-old clinical practices^24^,^25^,^26^. To some degree, the combinatorial roles of multiple active compounds were disregarded during the modern screening process. Therefore, the authors thought that drug discovery was not necessarily confined to single molecules. The shifting of screening “single bioactive compound” to “single bioactive compound, drug combination and multicomponent interaction” may make a significant difference in drug discovery.

Against the above background, a novel strategy was proposed to increase the compatibility between the NPLs and the screening technologies, which is helpful for promoting the hit rate of lead compounds in drug discovery. The current study also aims to establish a new mode for comprehensively exploring both the bioactive molecules and multicomponent interactions in herbal medicines, which might be the key to explanation of their pharmacological benefits. The general procedures of our strategy mainly include the following five steps as summarized in Fig. 1. (1) Classification of the compounds in an herb into several chemical families. (2) Reconstruction of a new compound library based on the original herb extract. (3) Mapping the bioactivity distribution and discovering the target chemical family. (4) Evaluation of multicomponent interactions from the inter- and intra-family perspectives. (5) Exploration of the potential mechanisms by *in silico* molecular docking and clustering analysis. A significant feature of this protocol was that the crude herbal extract was replaced with the reconstructed compound library for high-throughput screening. Compared with the conventional methods, this protocol avoided the time-consuming and labor-intensive purification of total reference standards, which would obviously decrease the cost of drug discovery. Based on the reconstruction theory, this strategy could also be expanded to other libraries containing compounds with the similar chemical skeletons such as combinatorial library.

As an illustrative case study, thrombin and Dan-Qi pair (DQP) were used as the experimental materials. Thrombin, an enzyme which plays a significant role in thromboembolic disease^27^, has been proved to be a target in the prevention of cardiovascular disease. DQP, containing an herb pair of Radix Salvia miltiorrhiza (Danshen in Chinese) and Radix Panax notoginseng (Sanqi in Chinese), has been widely used for the treatment of cardiovascular diseases in China since ancient times^28^. According to the clinical experience of DQP, it is reasonable to assume that some bioactive components against thrombin may exist and there are significant interactions among multi-components. Thus, DQP was used as the natural product library for illustrating the present strategy.

## Results

### Optimization of HPLC fingerprint and classification of the peaks

An optimum fingerprint that each peak can be baseline separated is of great importance for the following peak-based fractionation and library reconstruction. Therefore, some important factors such as chromatographic columns, the composition of the mobile phase and the elution program were systematically explored. Finally, steady baselines and good peak shapes were clearly seen in both the analytical chromatogram (Figure S1a) and the semi-preparative chromatogram (Figure S1b) with the elution conditions described in Methods. Based on the optimized elution program, HPLC-Q-TOF-MS/MS was conducted to identify the compounds in DQP extract. By comparing retention time (t_R_) and characteristic fragmentation ions with those of the reference compounds and the data in literature, a total of 18 compounds were unambiguously identified as shown in Table 1.

**Figure 2:**
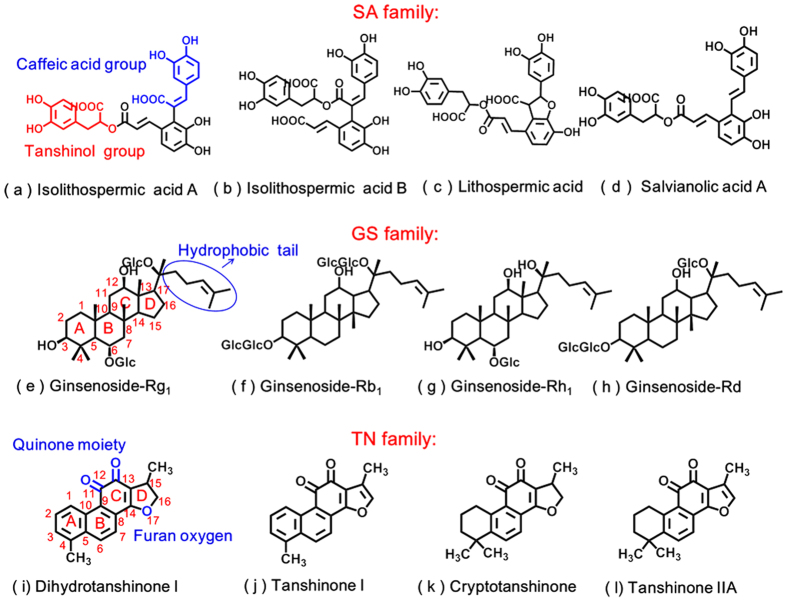
Representative compounds and structural characteristics of SA, GS and TN families in DQP. SA family includes (**a**) isolithospermic acid A, (**b**) isolithospermic acid B, (**c**) lithospermic acid and (**d**) salvianolic acid A; SAs are polymers of caffeic acid (C_9_H_8_O_4_) and tanshinol (C_9_H_10_O_5_), of which carboxyl and phenol hydroxyl are two characteristic groups. GS family includes (**a**) ginsenoside-Rg1, (**b**) ginsenoside-Rb1, (**c**) ginsenoside-Rh1 and (**d**) ginsenoside-Rd; GS has a nucleus with 17 carbon atoms arranged in four rings, to which the hydrophobic tail structure and hydrophilic sugar moieties are attached. TN family includes (**a**) dihydrotanshinone I, (**b**) tanshinone I, (**c**) cryptotanshinone and (**d**) tanshinone IIA; TN also possesses a similar 17-atom skeleton, but the quinone moiety in the C-ring, furan oxygen in the D-ring and numerous conjugated double bonds make it different.

**Figure 3:**
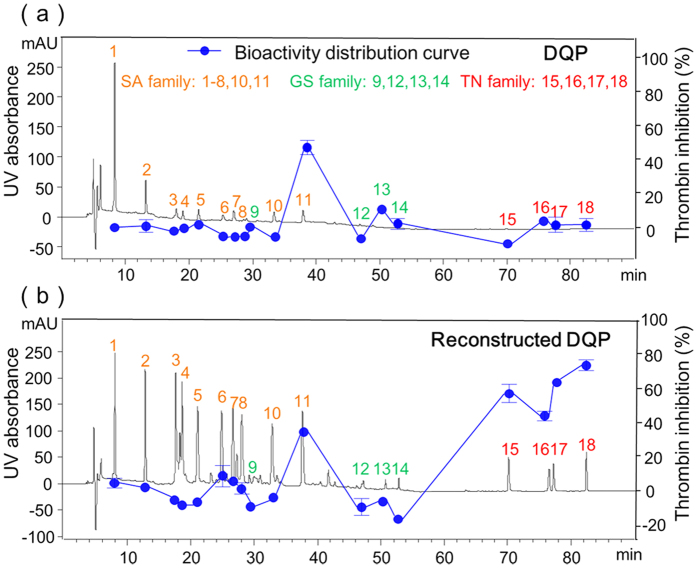
Chromatographic fingerprint-bioactivity maps of (**a**) DQP and (**b**) reconstructed DQP. The 18 peaks in DQP were assigned to three chemical families as follows: peaks 1–8, 10 and 11 belonged to SA family; peaks 9, 12, 13 and 14 belonged to GS family; peaks 15, 16, 17 and 18 belonged to TN family.

The 18 compounds in DQP were divided into three families, namely, salvianolic acid (SA), ginsenoside (GS), and tanshinone (TN). The representative compounds are shown in Fig. 2. SAs are polymers of caffeic acid (C_9_H_8_O_4_) and tanshinol (C_9_H_10_O_5_), where carboxyl and phenol hydroxyl are two characteristic groups; GS has a nucleus with 17 carbon atoms arranged in four rings, to which the hydrophobic tail structure and hydrophilic sugar moieties are attached; TN also possesses a similar 17-atom skeleton, but the quinone moiety in the C-ring, furan oxygen in the D-ring and numerous conjugated double bonds make it different. Correspondingly, the peaks in the chromatogram of DQP were assigned as follows (Fig. 3a): peaks 1–8, 10 and 11 belong to SA family; peaks 9, 12, 13 and 14 belong to GS family; peaks 15, 16, 17 and 18 belong to TN family.

### Peak-based fractionation and library reconstruction

In the present study, the DQP extract contained 18 compounds, of which the contents ranged from 0.05% to 2.67% according to our previous quantitative results^21^. Due to the considerable content difference among the 18 compounds, it was not suitable to directly use the DQP extract as the screening library. Thus, we decided to reconstruct a new library by peak-based fractionation and recombination. A typical semi-preparative chromatogram of the DQP extract is shown in Figure S1b, and the corresponding collection program based on the time window of each peak is exhibited in Table S1. Because the different collection volumes of each peak would bring trouble to the calculation for library reconstruction, a high-throughput vacuum centrifugal evaporator was applied to remove the solvents, and then 200 μL of methanol were added to each fraction. These redissolved fractions would be used to reconstruct the new compound library. As previously discussed, the 18 compounds in DQP were assigned to three chemical families including SA, GS and TN. Three commercially available reference compounds, namely, salvianolic acid A, ginsenoside-Rh1 and tanshinone IIA, were used as the representatives of the above three families for normalizing the other family members. Their peak areas at the concentration of 100 μM were 2302, 269 and 787, respectively. The reason for choosing 100 μM is that the bioactivity at this concentration could be used to preliminarily estimate the potential value of a compound and determine whether to conduct in-depth research in enzymatic activity assay. Followed by a series of calculations and peak recombination, a new library was generated and the chromatogram is shown in Fig. 3b. The areas of peaks in one family were at the same level and almost equal to the standards (Table S2): the area of SA ranged from 2044 to 2601; the area of GS ranged from 218 to 284; the area of TN ranged from 721 to 783. By this way, we obtained a reconstructed compound library derived from DQP, in which the compounds were unambiguously identified and the concentration of each compound was relatively clear (close to 100 μM). More detailed procedures and formula derivation can be seen in Methods.

### Screen for thrombin inhibitors by reconstructed chromatographic fingerprint-bioactivity map

Chromatographic fingerprint–bioactivity map is an emerging approach to discovering lead compounds from herbal medicines^29^,^30^,^31^. It does not require commercially expensive reference compounds or much organic solvent for multi-step isolation, and thus it is green, simple and economical. However, the main disadvantage of this method is that although the bioactivity distribution in chromatographic fingerprint could be observed, the absolute quantity of the compound contained in each peak is unknown, leading to the false-negative results for some minor compounds. To solve the problem, the previously reconstructed library was combined with chromatographic fingerprint–bioactivity map to screen bioactive compounds, in which the concentration of each compound was normalized to the same level and relatively clear. Actually, the new method was “reconstructed chromatographic fingerprint-bioactivity map”.

Figure 3b shows the distribution of thrombin inhibitory activity in the reconstructed chromatographic fingerprint of DQP. It clearly suggested that peaks 11, 15, 16, 17 and 18 exhibited thrombin inhibitory effect, among which peak 11 belongs to the SA family and the other 4 peaks are from TN family (Table 1). The results implied that TN family might be an important class of thrombin inhibitors. Moreover, the conventional method of chromatographic fingerprint–bioactivity map was also conducted as a comparison. As shown in Fig. 3a, peak 11 exhibited thrombin inhibition, but the minor peaks of 15, 16, 17 and 18 seemed inactive. By comparing Fig. 3a,b, it was easily observed that the difference was the four peaks in TN family. To explore whether the four TNs were bioactive or not, we obtained the corresponding reference compounds from the National Institute for the Control of Pharmaceutical and Biological Product, and then conducted enzymatic activity assays. The final results suggested that dihydrotanshinone I (peak 15), cryptotanshinone (peak 16), tanshinone I (peak 17) and tanshinone IIA (peak 18) possessed thrombin inhibitory activity with IC_50_ values of 92, 102, 333 and 39 μM, respectively (Table 2), demonstrating that the new method of reconstructed chromatographic fingerprint-bioactivity map had obvious advantage in screening minor compounds. Interestingly, the inhibition rate of salvianolic acid A (peak 11) was merely −0.82% at a concentration of 125 μM (Table 2), showing that it did not possess thrombin inhibitory ability, which was contradictory to the result of reconstructed chromatographic fingerprint-bioactivity map. Considering salvianolic acid A was unstable and easily transformed to other analogues in phosphate buffer solution (PBS)^32^, we assumed that salvianolic acid A inhibited thrombin by its transformed products, and conducted an experiment to verify it. Salvianolic acid A was dissolved in PBS and the solution was tested at different points of time. As shown in Figure S2, the inhibitory ratio of the test solution increased with the time of salvianolic acid A staying in PBS, indicating that the transformation process played an important role in thrombin inhibition.

### Identification of intra-family interactions among TNs

**Figure 4:**
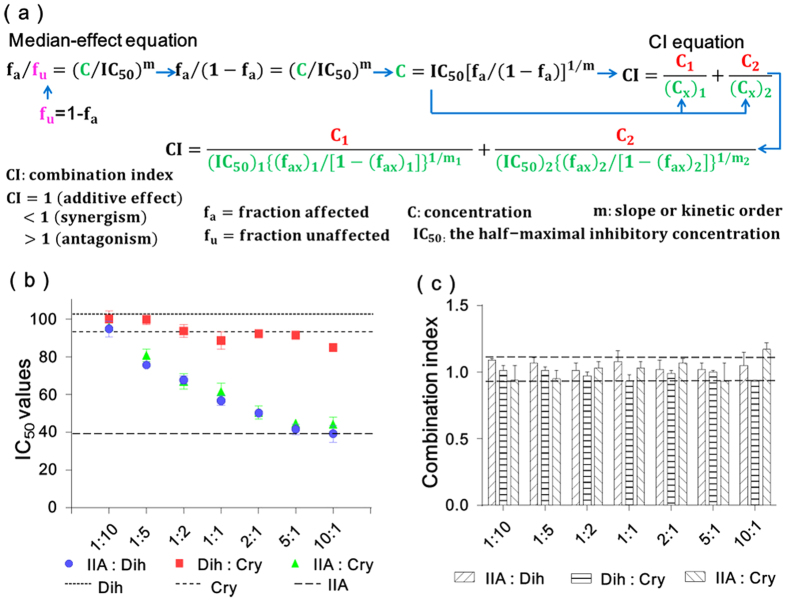
Identification of the interactions among tanshinones (TNs). (**a**) Algorithms for calculating the combination index (CI) derived from the median-effect equation. (**b**) IC50 values of different TN pairs at different combination ratios. TNs included dihydrotanshinone (Dih), tanshinone IIA (IIA) and cryptotanshinone (Cry). (**c**) The CI values of the three TN combinations at different ratios.

To determine compound-compound interactions and discover candidate drug pairs in TN family, the combination index (CI) coupled with an enzymatic activity assay was adopted. The CI theorem, which is derived from the mass-action law principle, has been widely used in researching drug combinations for treating diseases such as cancer and AIDS^33^. In the present work, the algorithm for evaluating compound-compound interactions in enzyme models is deduced by merging the median-effect equation and the CI equation (Fig. 4a), which offers quantitative definition for additive effect (CI = 1), antagonism (CI > 1), and synergism (CI < 1)^34^,^35^. Detailed illustration about the algorithm can be seen in Methods. As shown in Fig. 4a, the IC_50_ values for single compounds alone and multiple compound-compound combinations are necessary for calculating CI values. Considering the much higher IC_50_ value and the poor solubility of tanshinone I (Table 2), it was obviously supraphysiologic and not suitable to combine with other compounds for application. Thus, this investigation would focus on the TN pairs combined by the other three TNs, namely, tanshinone IIA-dihydrotanshinone I, dihydrotanshinone I-cryptotanshinone, and tanshinone IIA-cryptotanshinone. To fully assess the interactions, a serial of 7 content ratios spanning from 1:10 to 10:1 were designed and the IC_50_ at each content ratio was assayed (Tables S3–S5). As shown in Fig. 4b, the IC_50_ values of each combination at different ratios were almost between those of the two single compounds, and thus no obvious antagonism and synergism could be observed. By software simulation, most of the CI values located in the range of 0.9 to 1.1 (Fig. 4c), suggesting that the interactions of the above three TN pairs were all additive effects. Obviously, the highly similar core structures and pharmacophores of TNs were the fundamental reason for this effect (Fig. 2), which implied that herbal medicines might function by a chemical family rather than a single compound. Moreover, the additive effects of the investigated TN pairs were relatively stable at various combination ratios, showing that they had potential research value in multicomponent therapeutics. These results would also be helpful for the further exploitation of DQP.

### Investigation of inter-family interactions among SA, GS and TN

There were three chemical families including SA, GS and TN in DQP, of which TN was the family with thrombin inhibition activity. To our surprise, the other two families showed totally different effects. As shown in Table 2, when the test concentration of each compound was 125 μM, the four members of GS family including ginsenoside-Rg1, ginsenoside-Rh1, ginsenoside-Rb1 and ginsenoside-Rd promoted thrombin by 6.73%, 9.24%, 13.70% and 11.64%, respectively. To exclude the possibility that the promotion effects were false results caused by experimental errors, we have tested GS at a series of gradient concentrations. The results suggested that the thrombin promotion effect of GS was stable and repeatable. However, the members of SA family were demonstrated to be inactive even at a very high concentration. Due to the completely different activities of the three families, it was interesting for us to investigate the inter-family interactions.

**Figure 5:**
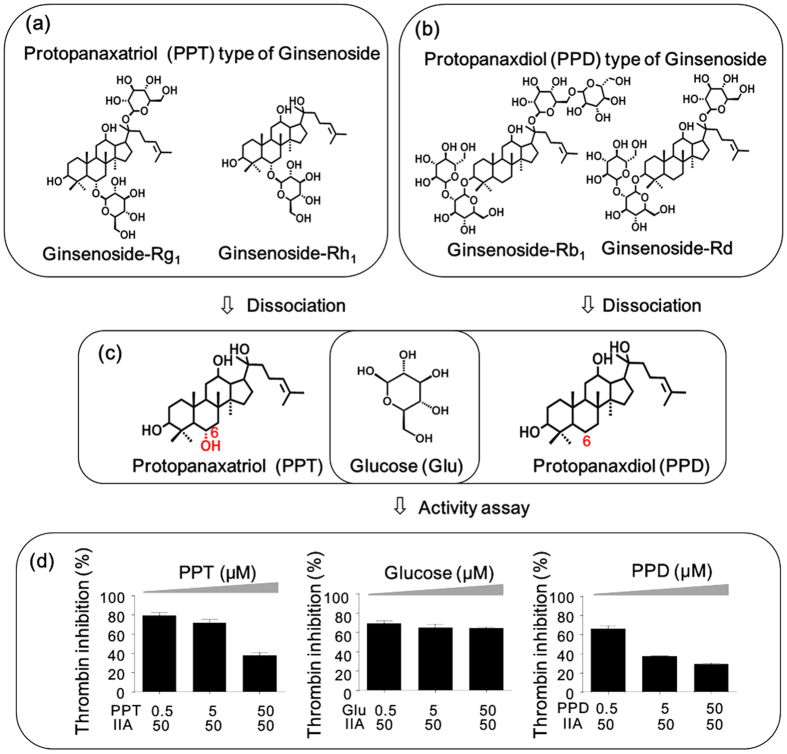
Investigation of the active core of GS that antagonized the TN-induced thrombin inhibition. (**a**) the structures of PPT-type GS; (**b**) the structures of PPD-type GS; (**c**) the three basic structures including PPT, PPD and glucose dissociated from GS; (**d**) the effect of PPT, PPD and glucose on TN-induced thrombin inhibition.

Because the effects of GS and TN on thrombin were opposite, the GS-TN interactions were firstly explored. Actually, the four members in GS family could be further divided into two sub-families according to the difference of aglycones, namely, PPT-type GS (Fig. 5a, Rg1 and Rh1) and PPD-type GS (Fig. 5b, Rb1 and Rd). PPT and PPD were the abbreviations of protopanaxatriol and protopanaxadiol, respectively. The former is distinguished from the latter by the presence of a hydroxyl at C-6 (Fig. 5c). As shown in Table 3, PPT-type GS reduced the TN-induced thrombin inhibitory effect in a dose-dependent manner, suggesting that the interactions between them were antagonism. Compared with PPT-type GSs, PPD-type GSs were more powerful: when the concentration of GS was equal to that of TN (1GS + 1TN), PPD-type GSs (Rd and Rb) could counteract or even reverse the TN-induced thrombin inhibition (Table 4). Different from the antagonism between TN and GS, SA almost has no dose-dependent influence on the thrombin inhibition of TN (Table S6a-b). Therefore, using thrombin as the connecting node, the interactions among TN, GS and SA were obtained: TN inhibited thrombin; GS promoted thrombin; GS antagonized TN-induced thrombin inhibition; SA had no influence on thrombin. To provide a better understanding of the above interactions, the typical kinetics of TN, GS, SA, GS-TN and TN-SA on thrombin were compared as shown in Figure S3. Based on these findings, the complex inter-family relationships in DQP were uncovered, which demonstrated that multicomponent interactions were non-ignorable in herbal medicines.

### Core structure of GS responsible for the antagonism

GS could antagonize the TN-induced thrombin inhibition, but its core structure responsible for the antagonism was unclear. Generally, a GS consists of the non-polar aglycone moiety and the polar sugar moiety (Fig. 5a,b), where the aglycone is used as the backbone and the sugars are conjugated to it. By dissociating the GSs in DQP, three fundamental substructures constituting GSs were obtained, namely, PPD, PPT and glucose (Fig. 5c). To determine the core structure responsible for the antagonism, it was necessary to assay the three compounds and evaluate their contribution in GS. The results are shown in Fig. 5d. As the concentration of PPT increased, the TN-induced thrombin inhibition decreased in a dose-dependent manner, indicating that PPT moiety contributes to the antagonism effect of GS. Similar result was obtained by PPD, which confirmed that the aglycones played an important role in GS. Different from the results of PPD and PPT, as the concentration of glucose increased, the TN-induced thrombin inhibition did not change and no antagonism effect was observed. These findings clearly suggested that the structurally active core of GS was the aglycone moiety rather than the sugar moiety.

It is worth noting that, compared with the antagonism of PPD, PPD-type GS counteracted or even reversed the TN-induced thrombin inhibition (Table 4), showing that the activity of PPD-type GS was more potent than that of the aglycone PPD. In other words, sugar moiety could enhance the activity of GS, although it was not the active core. This conclusion was supported by another result that PPD-type GS with more sugar moieties (Table 4) exhibited greater activities than PPT-type GS (Table 3).

### Exploration of the mechanisms for multicomponent interactions

To investigate the potential mechanisms behind the multicomponent interactions in DQP, an *in silico* molecular docking was performed. The related compounds were docked to the whole thrombin protein, and 100 possible conformations were generated. Correspondingly, the binding energy of each conformation, which reflects the binding affinity of ligand to thrombin, was obtained. To find the most possible binding site, we introduced clustering analysis to rapidly classify the 100 conformations based on the similarities and rank the potential sites according to the binding energies. Once the binding site was identified, the specific amino acid residues constituting this site would be exposed and further analysed. Using the above method, the four compounds in TN family were firstly explored. Interestingly, the clustering analyses showed that the binding sites of TNs were almost the same (Figure S4). By comparing the bonded residues with the literature^36^,^37^, this site was determined as the active pocket of thrombin for catalysing substrate hydrolysis. Therefore, it was rational to assume that TN inhibited thrombin by binding to the active site and disturbing its function. The results also suggested that the additive interactions among TNs were due to their same mechanism for thrombin inhibition, namely, binding to the same active pocket.

**Figure 6:**
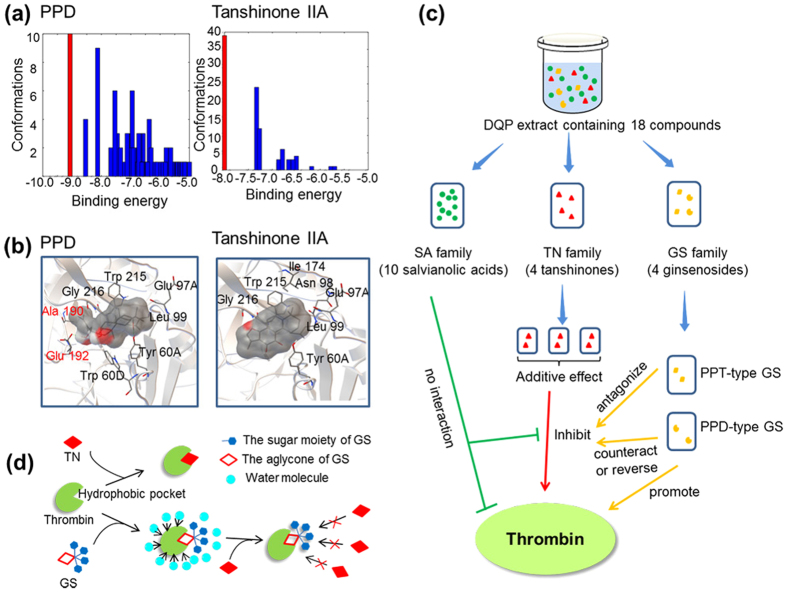
(**a**) Schematic representation of multicomponent interactions in DQP. (**b**) The histograms showed clustering analyses of PPD-thrombin conformations (left) and tanshinone IIA-thrombin conformations (right). (**c**) The interactions of PPD (left) and tanshinone IIA (right) to thrombin. (**d**) The potential mechanisms of TN inhibiting thrombin, GS promoting thrombin, and GS antagonizing TN-induced thrombin inhibition.

To explore the activity core of GS, the three basic structures of PPD, PPT and glucose constituting GSs were docked to thrombin. As shown in Figure S5, the binding position of glucose was far away from the active site, indicating that it was not the key structure of GS responsible for the antagonism. Notably, PPD and PPT could interact with the active site with relatively low binding energies, which exhibited the potential competitive relationship with TN. The above results of docking were consistent with those of enzymatic function assay (Fig. 5). To uncover the functional differences between TN and GS, two representatives of tanshinone IIA and PPD with the relationship of antagonism were used as the illustrative cases for comparison. As shown in Fig. 6a, the lowest binding energies of PPD and tanshinone IIA were −9.07 and −8.00 kcal/mol, respectively. The result suggested that PPD had a competitive advantage in preferentially binding to thrombin, which might be a premise and foundation of PPD antagonizing TN-induced thrombin inhibition. According to the reported crystal structure of thrombin, there are three principal binding pockets at the active site of thrombin, namely, the specificity (S) pocket, the proximal (P) pocket and the distal (D) pocket^36^,^37^,^38^,^39^. The bonded residues around PPD (Fig. 6b left) could be assigned to the above three pockets: (1) Ala 190 and Glu 192 belong to S pocket; (2) Trp 60D and Tyr 60A belong to P pocket; (3) Leu 99, Trp 215 and Gly 216 belong to D pocket. The bonded residues around tanshinone IIA (Fig. 6b right) could be assigned to two pockets: (1) Tyr 60A belongs to P pocket; (2) Leu 99, Trp 215 and Gly 216 belong to D pocket. By comparing the above information of PPD and tanshinone IIA, the difference was focused on S pocket. By carefully recognizing the spatial orientations of compounds in their conformation (shadows in Fig. 6b), the tail structure of PPD (Fig. 2) was found to be responsible for binding with S pocket. In other words, the four-ring nucleus of PPD contributed to the other two pockets, namely, P pocket and D pocket, which was consistent with that of tanshinone IIA due to the similar molecular shape. Because the tail is a characteristic structure of GS family, it was possible that GS promoted thrombin by influencing the function of S pocket. From another perspective, one obvious structural difference between TN and GS is that TN possesses numerous conjugated double bonds (Fig. 2). It has been demonstrated that the double bond was an important hydrophobic group for influencing the distribution of electron density and increasing the molecular hydrophobicity^40^,^41^. Interestingly, the three pockets constituting the active site of thrombin were also hydrophobic. Therefore, it was reasonable to assume that double bonds in TN contributed to forming hydrophobic interaction with the active site of thrombin. Once the stable ligand-enzyme complexes were generated, the function of thrombin would be inhibited. The hypothesis was supported by the structure-activity comparison of cryptotanshinone and tanshinone IIA (Figure S6): the IC_50_ of the later was 39 μM, much lower than 102 μM of the former; however, the only structural difference between them was a double bond in D ring.

## Discussion

Herbal medicines have gained increasing popularity worldwide because they have provided numerous leads or drugs for various diseases. It is well known that most herbal extracts are rather complex consisting of several major and a large number of minor compounds. The objectives of current screening methods are mainly focused on the major components rather than minor components because the later will cause a series of problems hindering deeper research^42^. However, there is no denying that numerous pharmacologically active molecules and drugs are originally from minor components in herbs such as paclitaxel and vincristine^43^. Screening of minor bioactive components from herbal medicines has been one of the bottlenecks in modern research^44^. Besides lead compounds, herbs can also provide potential drug combinations and useful multicomponent interactions, which had gradually formed during the long-term clinical practice in the ancient times. However, these aspects seem to be ignored or even abandoned in the modern screening. One important reason for this is the complexity of herbs: it has been difficult to discriminate the bioactive leads from a mixture, let alone the complicated and unpredictable compound-compound interactions.

In this work, we proposed a novel chemical family-based strategy to comprehensively uncover hidden bioactive molecules and multicomponent interactions in herbal medicines. “Chemical family” was the core concept in this strategy with the attempt to simplify current compound-based studies. We defined the “chemical family” as a group of compounds with similar skeletons and pharmacophores, which may possess similar physical, chemical and pharmacological properties. The rationale behind this concept is that, since herbs produce and accumulate several types of analogues in different pathways of secondary metabolism, each one has a common and characteristic nucleus. The concept of chemical family was the bridge between single compounds and multicomponent interactions in this strategy, which could simplify herbal medicines by several orders of magnitude. For instance, more than one hundred of compounds may exist in DQP, but there are only three chemical families, namely, SA, GS and TN.

From the view of chemical family, a library reconstruction method was designed to build a more suitable compound library for activity screening. In the ideal library, the chemical structure corresponding to each peak was clearly identified, and the contents of all compounds were normalized to a fixed concentration. Thus, when this library was combined with high-throughput screening technology, the results could accurately reflect concentration-effect relationship and avoid the false-positive or false-negative results to the utmost. This method involves two key procedures of peak-based fractionation and peak recombination. The peak-based fractionation was performed by semi-preparative RP-HPLC and the purpose was to accumulate enough raw materials for library reconstruction. The running times of RP-HPLC depends on the peak with the lowest content, which usually consumes a few hours to several days. In the following procedure of peak recombination, quantitative analysis of multi-components by single-maker (QAMS), a novel and rational method for quality control of herbal products, was used as the guideline and theoretical basis. The principle of this method is that a single reference compound could be used to directly determine the other compounds when the chemical structures and UV absorption of the analytes possess high similarities^45^. Inspired by that, we classified the compounds in an herbal extract into several chemical families based on the structural similarity, and choose one representative compound in each family to determine and normalize the others. Finally, library reconstruction was conducted by recombination of the collected peaks through a serial of QAMS-derived algorithms. After integrating the reconstructed library with high-throughput screening, the chemical families in the herb could usually be divided into the bioactive and the non-bioactive. Using the bioactive family as the clue, the complex intra- and inter-family interactions will be systematically explored. This method does not require the total reference standards, and it could also be coupled with other useful and complementary techniques such as bioassay-guided fractionation for bioactivity screening^46^,^47^.

Taking thrombin as the model enzyme, the bioactive molecules and multicomponent interactions in DQP were successful uncovered as shown in Fig. 6c. The compounds in DQP were assigned to three families of SA, GS and TN. Interestingly, they played completely different roles: SA almost had no effect on thrombin; TN could inhibit thrombin; GS showed the potential to promote thrombin. Among the above three families, TN exhibited the greatest value in treating thrombin-related diseases such as thrombosis, and thus the intra-family interactions among TNs were investigated. The results suggested that the members in TN family took effect in an additive mode. Using TN as the connecting point, the TN-SA interaction and TN-GS interaction were explored, respectively. On one hand, no significant interaction between TN and SA was observed. However, on the other hand, the interactions between TN and GS were relatively complex: PPT-type GS antagonized the TN-induced thrombin inhibition; PPD-type GS with more powerful ability could counteract or even reverse it.

Based on the results of docking and structural analysis, the antagonism between TN and GS could be clearly simulated by the famous hydrophobic-collapse model^48^,^49^. In this model, hydrophobic interaction plays a key role in protein folding: non-polar amino acid residues are driven from water molecules and buried in protein interior, while the polar residues are exposed on protein surface, leading to the collapse of the protein into a globular state^50^. Thrombin was a typical representative of this model, in which the pockets at the active site were hydrophobic and shielded from the surrounding solvent of water. As shown in Fig. 6d, when TN encountered thrombin in water, due to the good shape complementarity between TN and thrombin, and the repulsion between TN and water, TN could rapidly bind to thrombin through hydrophobic interaction. In this process, double bonds in TNs played important roles in keeping TN-thrombin complexes stable and further inducing thrombin inhibition. When GS bound to thrombin, the aglycone moiety interacted with the active site and the hydrophilic sugar moiety pointed outward. GS seemed to be a mask over the active site of thrombin, which could reduce the driving force of water. As a result, the functional residues of the active site were exposed and the activity of thrombin was promoted. Because of the competitive relationship on the active site, GS could block the binding of TN to thrombin, resulting in the antagonism between TN and GS. Moreover, it can be estimated that the outward polar sugar moiety, which could repel the hydrophobic TN, was beneficial to enhance the antagonism effect of GS. This hypothesis was verified by the structure-activity relationship that GS with more sugar moieties showed a stronger antagonism to TN (Table 3, 4). These results demonstrated the hydrophobic-collapse model was helpful for better understanding of multicomponent interactions in DQP.

In conclusion, this study reported a novel strategy for easier and deeper screening of herbal medicines. All these results introduced by this strategy, namely, screening for the minor bioactive compounds, discovering the interesting compound combinations and elucidating the multi-component interactions could be applied to discover new drug leads, control the quality of herbs and understand the herbal pharmacological effects. The newly established method of library reconstruction is a useful approach to increase the compatibility between high-throughput screening and compound libraries such as herbal extracts, food products and combinatorial libraries. Because it consumes only several reference standards and does not require the multi-step purification, the practical and economical method is applicable in general laboratories. Using this strategy, the multicomponent interactions in DQP were comprehensively uncovered for the first time. It is worth noting that the interaction between TN family and GS family was antagonism, of which the degree was adjustable by using different types of GS. This finding has a great potential value in designing targeted drug system and target-missing system in pharmaceutics. Moreover, the present work also provided three new and promising TN pairs for thrombin-related diseases such as thrombosis, which were demonstrated to possess stable additive effects over a wide range of combination ratio. The proposed strategy was expected to be a promising screening mode for herbal medicines and make a significant difference in drug discovery. Our future studies will focus on method development with multiple targets, discovery of synergistic combinations and optimization of screening models.

## Methods

### Materials and reagents

DQP preparation extracted from Radix Salvia miltiorrhiza and Radix Panax notoginseng was generously provided by Tianjin Tasly Pharmaceutical Co. Ltd. (Tianjin, China). Thrombin (E.C. 3.4.4.13) from bovine plasma was purchased from Shenyang Baiying Co. Ltd. (Shenyang, China). The chromogenic substrate for thrombin S-2238 was purchased from Adhoc International Technologies Co. Ltd. (Beijing, China). Argatroban was obtained from Sigma-Aldrich (St. Louis Missouri, USA). The reference standards of tanshinol, protocatechuic aldehyde, rosmarinic acid, lithospermic acid, ginsenoside-Rg1, salvianolic acid B, salvianolic acid A, ginsenoside-Rb1, ginsenoside-Rh1, ginsenoside-Rd, dihydrotanshinone I, tanshinone I, cryptotanshinone and tanshinone IIA were purchased from the National Institute for the Control of Pharmaceutical and Biological Product (Beijing, China). Isolithospermic acid A, isolithospermic acid B, salvianolic acid D and salvianolic acid G were previously isolated and structurally identified in the authors’ laboratory. Distilled-deionized water was provided by a Milli-Q water purification system from Millipore (Bedford, MA, USA). Formic acid and acetonitrile (HPLC grade) were purchased from Merck (Darmstadt, Germany).

### Structural identification of compounds in DQP

An Agilent 1200 SL rapid resolution system equipped with a binary pump, an online degasser, an auto-sampler, a thermostatically controlled column compartment and a 6520 Q-TOF mass spectrometer containing an electrospray ionization (ESI) source (Agilent Technologies, Santa Clara, CA, USA) was applied to analyze DQP. The chromatographic separation was performed on an Agilent Zorbax Extend-C18 column (250 × 4.6 mm, 5 μm) and a C18 guard column. The mobile phase consisted of 0.02% formic acid in water (A) and 0.02% formic acid in ACN (B) with a flow rate of 0.5 mL/min at room temperature. The gradient elution program was 10–21% B at 0–9 min, 21–22% B at 9–18 min, 22–24% B at 18–23 min, 24–27% B at 23–33 min, 27–33% B at 33–40 min, 33–41% B at 40–52 min, 41–70% B at 52–64 min, 70–72% B at 64–72 min, 72–100% B at 72–80 min, 100–100% B at 80–90 min. The UV detection was 203 nm for 0–56.5 min and 281 nm for 56.5–90 min. The ESI source of mass spectrometer worked in both the negative ion and positive ion modes. The operating parameters were set as follows: drying gas temperature, 325 °C; drying gas (N_2_) flow rate, 10.0 L/min; nebulizer, 35 psig; fragmentor voltage, 120 V; capillary, 3500 V; OCT RF V, 250 V; skimmer voltage, 65 V. For MS/MS experiments, three collision energies of 20, 40 and 80 V were applied to obtain maximal structural information. The mass range was set at m/z 100–1000. The acquisition and analysis of data were performed under Masshunter Workstation Software (version B.02.00). After the structures of compounds in DQP were identified, all compounds would be classified into several chemical family based on their structural characteristics, pharmacophores and molecular weights.

### Peak-based fractionation and library reconstruction

To prepare the sample solution for peak fractionation, 200 mg of DQP was dissolved in 1 mL of water. The solution was centrifuged at 13,000 rpm for 10 min, and the supernatant was added to auto-sample vials for injection. The instrumental system for peak fractionation was performed on an Agilent series 1100 HPLC system (Agilent, Germany) equipped with a binary pump, an online degasser, an auto-sampler, a column oven and a variable wavelength detector (VWD). Chromatographic separation was operated on a semi-preparative Zorbax SB-C18 column (9.4 × 250 mm, 5 μm, Agilent, USA) at 25 °C. Mobile phase consisted of water containing 0.2% formic acid (solvent A) and acetonitrile (solvent B). Sample solution was eluted by the following program at the flow rate of 2 mL/min: 10–22% B at 0–10 min, 22–23% B at 10–18 min, 23–24% B at 18–23 min, 24–27% B at 23–33 min, 27–33% B at 33–40 min, 33–41% B at 40–52 min, 41–70% B at 52–54 min, 70–72% B at 54–62 min, 72–100% B at 62–70 min, 100% B at 70–77 min. For segmental monitoring based on UV, different detection wavelengths were performed for different periods of time: 203 nm for 0–60 min; 281 nm for 60–77 min. The injection volume was 20 μL.

A total of 18 peaks classified into three families, namely, salvianolic acid (SA), ginsenoside (GS) and tanshinone (TN), were collected. The peak fractions were dried in a Genevac EZ-2 Evaporator vacuum centrifuge (Genevac Inc., New York, USA) at 50 °C, and the drying process was completed within 5 h. The residue of each fraction was dissolved in 200 μL of methanol. Three compounds salvianolic acid A, ginsenoside-Rh1 and tanshinone IIA were used as the standards to determine their corresponding families SA, GS and TN, respectively. The peak area of 100 μM standard in each family was used as the normalized criteria, and the volumes of the other compounds in the family will be calculated to make their final areas close to the criteria. Finally, the 18 peaks were recombined according to a series of algorithms, and the mixture was injected to HPLC for validation of peak areas. The theoretical basis and specific algorithms are as follows.

Step 1: Identification of compounds in herbal medicines and classification of them into chemical families. For example, there were 18 compounds in DQP and their corresponding peak areas were recorded as A_1_, A_2_, A_3_…A_18_. According to the structural similarity, they were classified into SA, GS and TN:


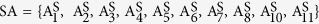



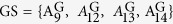



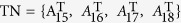


Step 2: Choose a representative compound from each chemical family and test the peak area at a certain concentration. These representatives are usually commercially available or easily prepared, and the certain concentration is a lead concentration to test the bioactivity. Specifically, three reference standards of salvianolic acid A, ginsenoside-Rh1 and tanshinone IIA were chosen from SA, GS and TN respectively, whose peak areas at the concentration of 100 μM were recorded as 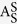, 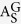 and 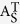 correspondingly. The three peak areas would be the standards for library reconstruction, and the theoretical basis was quantitative analysis of multi-components by single-maker (QAMS) as follows.

The response factor (f_i_) is defined as


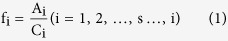


where A_i_ is the peak area, and C_i_ is the corresponding concentration. Using compound s as the standard, the relative response factor (f_si_) can be expressed as


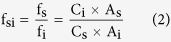


Furthermore, the equation which can be used to quantify the other analytes is deduced:


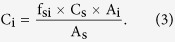


QAMS method with conversion factors was not widely used in Chinese Pharmacopoeia 2010 and European Pharmacopoeia 7.0 because of the potential fluctuation of relative response factor (f_si_) in different laboratories. Nevertheless, when the analytes possess highly similar structures and optical absorption, f_si_ can be considered as the value of 1. Therefore, the equation (3) could be simplified as 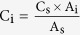 when the analytes were a series of analogues. This method was adopted to control the quality of Cat’s Claw in United States Pharmacopeia 33 and Rhizoma Coptidis in Chinese Pharmacopoeia 2010. In the present method of library reconstruction, the peak area (A_s_) and concentration (C_s_) of the standard are known; as long as the peak area (A_i_) of each compound is adjusted to be equal to A_s_, the corresponding concentration will be normalized to C_s_.

Step 3: Calculate the number (Y) of HPLC runs needed for the library reconstruction as equations (4, 5, 6). The maximum number (Y_max_) is usually dependent on the components with the low contents. If the Y_max_ is 100, it is impossible to run HPLC 100 times to collect the materials for reconstruction because it was time-consuming and labor-intensive. For this reason, the process will be scaled up by using a semi-preparative column with the same chromatographic packing. By increasing the injection volume or the sample concentration to 100 fold, this step could be completed with a single run, which is time-saving, effortless and environment-friendly.


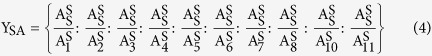



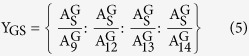



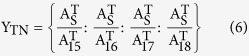



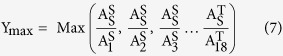


Step 4: The peak fractions are recombined according to the ratios described in step 3. In this way, a new compound library is generated, in which the compound contents are relatively uniform. The above steps apply to the peaks satisfying Gaussian distribution (normal distribution) because it is the basis of quantitative analysis. In fact, influenced by various factors such as pH of mobile phase, some peaks cannot meet 100% of Gaussian distribution, leading to the arising of experimental errors. In this case, it is necessary to adjust the ratios for peak recombination. The principle is to increase the proportion of a component when its peak area is less than the standard area, and vise versa. The degree of adjustment depends on the specific situation.

### Thrombin inhibitory activity assay

The thrombin inhibition studies were carried out in a 96-well microplate reader (Synergy 2, Bio-Tek Instrument Inc., Winooski, VT, USA) with the modified method^51^. The principle is that thrombin can catalyze the hydrolysis of chromogenic substrate S2238 (H-D-Phe-Pip-Arg-pNA·2HCl) and the process is detectable at 405 nm. The reaction mixture containing 100 μL of test solution and 75 μL of thrombin solution (0.5 U/mL) were added to each well and incubated for 15 min at room temperature. To initiate the chromogenic reaction, 25 μL of 150 μM chromogenic substrate was added to each well by means of a multichannel pipette to ensure that all reactions began simultaneously. The absorbance was detected at 405 nm every 20 s for 15 min. In the blank control group, the same volume of 1.2% DMSO phosphate buffer solution (75 mM, pH 7.4) was added instead of a solution of test compound. Argatroban, a known thrombin inhibitor, was used as the positive control. All experiments were done in triplicate and inhibition percentages were shown as mean values of triplicate observations. The thrombin inhibition (%) was calculated as inhibitory ratio = (1-α/β) × 100, where α is the linear change in absorbance per minute of the test solution, and β is the linear change in absorbance per minute of the blank control. The IC_50_ values of related compounds were determined on three replicates of 10 concentrations using the CompuSyn software, namely, 400, 200, 100, 50, 25, 12.5, 6.25, 3.13, 1.56 and 0.78 μM.

### Explore the interactions among TNs

The experiments were conducted with reference compounds obtained from National Institute for the Control of Pharmaceutical and Biological Product. Assay of the dose-effect curve and IC_50_ value of each TN was a prerequisite step for evaluation of combination effects. We explored three pairs, namely, tanshinone IIA with dihydrotanshinone I, dihydrotanshinone I with cryptotanshinone, and tanshinone IIA with cryptotanshinone, to illustrate the interactions among TNs. For each pair, a serial of 7 content ratios including 1:10, 1:5, 1:2, 1:1, 2:1, 5:1 and 10:1 were designed to assess the potential interactions. The dose-effect curves of the three pairs at 7 ratios in multiple diluted concentrations were plotted.

A combination index (CI) method that provides qualitative information on the nature of compound interaction (antagonistic, additive or synergistic effect) was used to analyze the results^52^. The theoretical basis of CI is the median-effect principle derived from numerous equations of different enzyme kinetic models in the presence of an inhibitor, demonstrating that there is a specific relationship between concentration and effect independently of mechanism, substrates and products^33^,^35^. In the model of enzyme reaction, the median-effect equation could be described as:


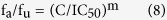


where f_a_ and f_u_ are the fractions affected and unaffected, respectively. For example, if thrombin is inhibited by 60%, f_a_ is 0.60 and f_u_ is 0.40 (f_u_ = 1 − f_a_). C is the concentration, IC_50_ is the half-maximal inhibitory concentration, and m is the slope of the median-effect plot denoting the shape of the dose-effect curve. When m is more than, equal to or less than 1, dose-effect curve is sigmoidal, hyperbolic or negative sigmoidal, respectively. Taking both the potency (IC_50_) and shape (m) into consideration, the method is more appropriate for evaluating compound interaction. If equation (8) was transformed, then:


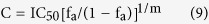


Based on a given effect (f_a_), the corresponding concentration (C) could be calculated when the m value and IC_50_ are determined. Therefore, the median-effect plot of y = log (f_a_/f_u_) versus x = log (IC_50_) deduced from the logarithmic form of Eq. (8) is applied, of which the x-intercept gives log (IC_50_) and the slope is m. Then, the calculated concentrations of the compounds and their combinations required to produce different levels of inhibition were plugged into the combination index equation (10):


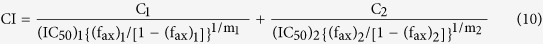


where CI is the combination index for two compounds at x% inhibition of thrombin. C_1_ is the portion of compound-1 in combination C_1_ + C_2_ that produces x% inhibition; correspondingly, C_2_ is the portion of compound-2 in combination C_1_ + C_2_ that produces x% inhibition. 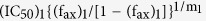
is the concentration of compound-1 alone that produces x% inhibition; similarly, 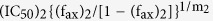
is the concentration of compound-2 alone that produces x% inhibition. Compound combinations having CI = 1 are considered as additive, those with CI < 1 are synergistic, and those with CI > 1 are antagonistic^53^.

### Investigation of inter-family interactions in DQP

To investigate inter-family interactions, we assayed the bioactivities of compound-compound pairs involved the chemical families of SA, GS and TN. In this assay, the concentration of TN was fixed, and the three gradient concentrations (low, medium, high) of SA or GS were added to influence the thrombin inhibitory ability of TN. The specific concentration of TN was chosen from the range around the IC_50_ values, because this range was the most sensitive on the sigmoidal concentration-effect curve. Moreover, considering that the inhibitory ratio between 50% and 70% was ideal for investigating inter-family interactions, the concentrations of tanshinone IIA, cryptotanshinone, dihydrotanshinone I and tanshinone I were set as 50 μM, 100 μM, 100 μM and 400 μM, respectively. Correspondingly, the three gradient concentrations (low, medium, high) of GS or SA were set as 1/4, 1/2 and 1 fold of that of TN, respectively. The procedure of thrombin activity assay was the same as mentioned before.

### *In silico* molecular docking

AutoDock 4.2 program (The Scripps Research Institute, La Jolla, CA, USA) was operated according to the standard protocol as follows. (1) Prepare the coordinate files of the ligand and receptor using AutoDockTools. These files are termed PDBQT, which contains the information of atom types and atomic partial charges. PDB files of enzyme were downloaded from the protein data bank (PDB); AutoDockTools (ADT) or other professional software was used to create PDB files of ligands. Both of them could be transformed to PDBQT from traditional PDB files. (2) Create the grid parameter files and run AutoGrid. Before the docking process, a pre-calculated grid map which helps to accelerate the docking calculations is necessary. It consists of a three-dimensional lattice of regularly spaced points. Finally, AutoGrid generates two files with the extensions of “.xyz” and “.fld”. The former depicts the spatial extent of the grids in Cartesian space and the latter is actually a field file containing the grid maps. (3) Choose a suitable protocol for application. Several protocols such as simulated annealing and traditional genetic algorithms were alternative in AutoDock. In the present work, a Lamarckian genetic algorithm (LGA), one of the most efficient methods, was utilized to search the most favorable ligand binding orientations. (4) Create docking parameter files and run AutoDock. Three related files are necessary: first, PDBQT files for enzyme and ligand; second, the grid maps for each atom type of the ligand; third, a docking parameter file including the parameters and files for docking calculation. The coordinates for every generated conformation, along with information of interaction energies and clustering, would be written into the docking log file (.dlg). (5) Analyze the results of docking simulations. AutoDock provides a cluster analysis of different docked conformations, which is expressed as a histogram. For each conformation, binding energy and bonded residues will be recorded.

## Additional Information

**How to cite this article**: Song, H.-P. *et al.* A chemical family-based strategy for uncovering hidden bioactive molecules and multicomponent interactions in herbal medicines. *Sci. Rep.* **6**, 23840; doi: 10.1038/srep23840 (2016).

## Supplementary Material

Supplementary Information

## Figures and Tables

**Figure 1:**
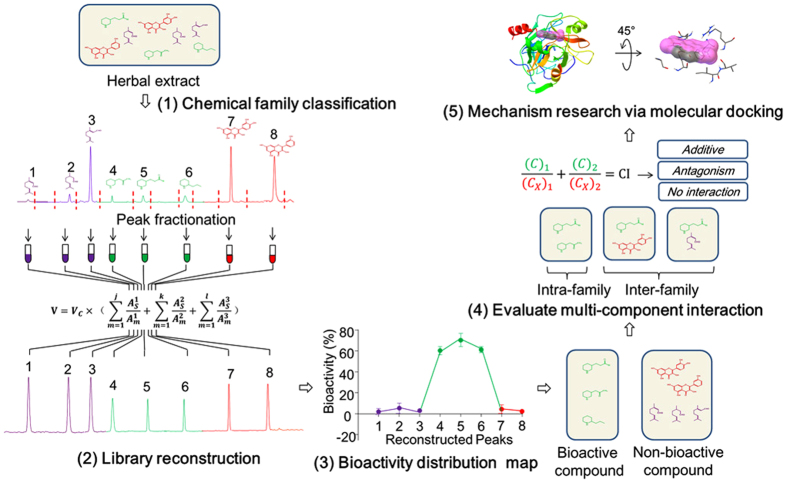
Diagram of the chemical family-based strategy for uncovering hidden bioactive molecules and multicomponent interactions in herbal medicines. The strategy mainly contains five steps: (**1**) Classification of the compounds in an herbal medicine into several chemical families. (**2**) Reconstruction of a new compound library based on the original herb extract. (**3**) Mapping the bioactivity distribution and discovering the target chemical family. (**4**) Evaluation of multicomponent interactions from the inter- and intra-family perspectives. (**5**) Exploration of the potential mechanisms by *in silico* molecular docking and clustering analysis.

**Table 1:** Retention time (t_R_), MS data and UV spectra for identification of 18 compounds in DQP by Q-TOF MS/MS.

Peak No.	t_R_ (min)	[M − H]^−^/[M + H]^+^		Diff. (ppm)	Fragment ions (m/z)	Elem. Comp.	Identification
		m/z	Calculate (m/z)				
1	8.32	197.0460	197.0455	−2.37	179.0351/135.0458	C_9_H_10_O_5_	Tanshinol
2	13.16	137.0242	137.0244	1.60	119.0143/108.0210	C_7_H_6_O_3_	Protocatechuic aldehyde
3	18.01	537.1043	537.1038	−0.88	493.1146/295.0615/185.0245/109.0293	C_27_H_20_O_12_	Isolithospermic acid A
4	19.00	537.1045	537.1038	−1.36	493.1143/295.0609/185.0244/109.0300	C_27_H_22_O_12_	Isolithospermic acid B
5	21.48	417.0828	417.0827	−0.23	197.0445/179.0344/135.0440	C_20_H_18_O_10_	Salvianolic acid D
6	25.37	339.0511	339.0510	−0.32	295.0618/293.0463	C_18_H_12_O_7_	Salvianolic acid G
7	27.00	359.0774	359.0772	−0.35	197.0456/179.0355/161.0239/135.0452	C_18_H_16_O_8_	Rosmarinic acid
8	28.60	537.1051	537.1038	−2.32	493.1138/313.0714/295.0612/197.0461	C_27_H_22_O_12_	Lithospermic acid
9	29.06	823.4793	823.4814	2.89	643.4121 /259.0312/203.0512/123.1618	C_42_H_72_O_14_	Ginsenoside-Rg1
10	33.40	717.1464	717.1461	−0.44	519.0926/493.1126/339.0514/321.0403/295.0611	C_36_H_30_O_16_	Salvianolic acid B
11	37.98	493.1138	493.1140	0.55	313.0740/295.0603/185.0251/203.0342	C_26_H_22_O_10_	Salvianolic acid A
12	46.89	1131.5921	1131.5922	0.04	789.4741/365.1048/425.3787/407.3675	C_54_H_92_O_23_	Ginsenoside-Rb1
13	50.31	–a	–	–	621.4359/423.3626/405.3508	C_36_H_62_O_9_	Ginsenoside-Rh1
14	52.36	969.5413	969.5393	1.00	767.4946/443.3876/425.3780/749.4824/407.3674	C_48_H_82_O_18_	Ginsenoside-Rd
15	69.95	279.1013	279.1016	0.97	261.0900/251.1057/233.0954	C_18_H_14_O_3_	Dihydrotanshinone I
16	76.25	297.1480	297.1485	1.50	279.1387/264.1142/251.1418	C_19_H_20_O_3_	Cryptotanshinone
17	77.03	277.0862	277.0859	−0.96	259.0754/249.0905/221.0958	C_18_H_12_O_3_	Tanshinone I
18	82.12	295.1313	295.1329	5.46	277.1208/249.1265/234.1047	C_19_H_18_O_3_	Tanshinone IIA

^a^The parent ion was not detected.

**Table 2:** Thrombin inhibition activity of the compounds in DQP.

Compounds	Chemical family	Inhibition (%)^a^	IC_50_ (μM)
Tanshinol	SA	4.13 ± 1.19	ND^b^
Protocatechuic aldehyde	SA	−1.49 ± 3.18	ND^b^
Rosmarinic acid	SA	−1.70 ± 0.54	ND^b^
Lithospermic acid	SA	−0.89 ± 0.83	ND^b^
Salvianolic acid B	SA	−0.91 ± 0.00	ND^b^
Salvianolic acid A	SA	−0.82 ± 1.88	ND^b^
Ginsenoside-Rg1	GS	−6.73 ± 1.01^c^	ND^b^
Ginsenoside-Rh1	GS	−9.24 ± 1.99^c^	ND^b^
Ginsenoside-Rd	GS	−11.64 ± 2.97^c^	ND^b^
Ginsenoside-Rb1	GS	−13.70 ± 3.71^c^	ND^b^
Dihydrotanshinone I	TN	63.71 ± 3.13	92 ± 5
Cryptotanshinone	TN	56.01 ± 2.21	102 ± 4
Tanshinone I	TN	39.79 ± 0.40	333 ± 28
Tanshinone IIA	TN	84.12 ± 1.81	39 ± 1
Argatroban	Positive control	99.46 ± 0.33	17 ± 1^d^

^a^The concentration of each reference standard was 125 μM.

^b^ND: not detected.

^c^The negative value means promotion effect on thrombin.

^d^The unit is nanomole.

**Table 3:** The interactions between TN and PPT-type GS for thrombin inhibitory activity.

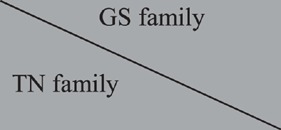	1.Ginsenoside-Rg1			2. Ginsenoside-Rh1		
	Thrombin inhibition (%) at different combination ratios			Thrombin inhibition (%) at different combination ratios		
	1/4GS + 1TN	1/2GS + 1TN	1GS + 1TN	1/4GS + 1TN	1/2GS + 1TN	1GS + 1TN
Dihydrotanshinone I	46.80 ± 3.19	41.76 ± 3.93	30.43 ± 0.72	38.03 ± 0.88	35.60 ± 0.51	26.58 ± 2.59
	Dose-dependent antagonism			Dose-dependent antagonism		
Tanshinone I	60.91 ± 0.63	51.25 ± 0.00	25.50 ± 12.68	51.19 ± 0.68	35.07 ± 8.73	25.12 ± 0.66
	Dose-dependent antagonism			Dose-dependent antagonism		
Cryptotanshinone	43.70 ± 1.28	33.70 ± 4.29	26.81 ± 2.70	61.3 ± 3.17	60.38 ± 2.52	7.54 ± 1.41
	Dose-dependent antagonism			Dose-dependent antagonism		
Tanshinone IIA	52.89 ± 0.74	47.28 ± 0.36	19.14 ± 1.79	34.12 ± 2.57	23.06 ± 1.36	17.57 ± 0.36
	Dose-dependent antagonism			Dose-dependent antagonism		

**Table 4:** The interactions between TN and PPD-type GS for thrombin inhibitory activity.

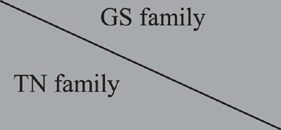	1. Ginsenoside-Rb1			2. Ginsenoside-Rd		
	Thrombin inhibition (%) at different combination ratios			Thrombin inhibition (%) at different combination ratios		
	1/4GS + 1TN	1/2GS + 1TN	1GS + 1TN	1/4GS + 1TN	1/2GS + 1TN	1GS + 1TN
Dihydrotanshinone I	44.30 ± 2.01	39.47 ± 3.72	9.87 ± 2.79	38.72 ± 1.92	27.61 ± 1.41	2.68 ± 2.37
	Counteraction^a^			Counteraction		
Tanshinone I	31.05 ± 8.19	30.19 ± 9.19	4.83 ± 4.83	71.20 ± 0.00	5.20 ± 3.39	2.20 ± 2.55
	Counteraction			Counteraction		
Cryptotanshinone	24.88 ± 0.86	−10.24 ± 2.41	−16.75 ± 3.44	8.82 ± 2.79	−5.31 ± 2.55	−7.65 ± 4.10
	Reverse^b^			Reverse		
Tanshinone IIA	34.02 ± 1.86	2.05 ± 0.32	−9.74 ± 0.48	69.17 ± 3.74	−2.09 ± 3.34	−4.10 ± 1.18
	Reverse			Reverse		

^a^The thrombin inhibition ranged from 0% to 10% when the combination ratio of GS and TN was 1:1(1GS + 1TN).

^b^The thrombin inhibition was negative when the combination ratio of GS and TN was 1:1(1GS + 1TN).
