# Supplementary material for: A chemical family-based strategy for uncovering hidden bioactive molecules and multicomponent interactions in herbal medicines
Source: Sci Rep. 2016 Mar 30;6:23840. doi: 10.1038/srep23840 (PMC4812296; doi:10.1038/srep23840)
Supplement: Supplementary Information [file srep23840-s1.pdf]

Supplementary Information

A chemical family-based strategy for uncovering hidden bioactive molecules and multicomponent interactions in herbal medicines

Hui-Peng Song*, Si-Qi Wu*, Haiping Hao, Jun Chen, Jun Lu, Xiaojun Xu, Ping Li & Hua Yang

State Key Laboratory of Natural Medicines (China Pharmaceutical University), No. 24, Tongjia Lane, Jiangsu, Nanjing 210009, China.

Correspondence and requests for materials should be addressed to P.L. (email: liping2004@126.com) or H.Y. (email: yanghuacpu@126.com)

* These authors contributed equally to this work.

1 Supplementary Figures

1.1 Fig. S1: The chromatographic profiles of DQP performed by analytical column (a) and semi-preparative column (b) …....………………...............4

1.2 Fig. S2: The thrombin inhibitory activity of salvianolic acid A increased with the time of dissolving in phosphate buffer solution……….………..……......4

1.3 Fig. S3: The kinetics of thrombin catalyzing substrate to product influenced by the representative compounds in different families........…...…………..5

1.4 Fig. S4: The docking results of dihydrotanshinone I, cryptotanshinone, tanshinone I and tanshinone IIA to thrombin…....………………………...…6

1.5 Fig. S5: The docking results of glucose (Glu), protopanaxadiol (PPD) and protopanaxatriol (PPT) to thrombin…..…………..………..…………......7

1.6 Fig. S6 The structure-activity comparison of cryptotanshinone (a) and tanshinone IIA (b) ………………….……………….………..…………......8

2 Supplementary Tables

2.1 Table S1：Collection window, collection volume and compound identification of 18 peak fractions……………………….....……………...……...…....9

2.2 Table S2: Comparison of the peak areas in DQP extract and the reconstructed library.……………………...……...…………………….……………….…....10

2.3 Table S3: Additive thrombin-inhibitory effects (IC_{50}, μM) of tanshinone IIA and dihydrotanshinone I..……………...………..……..……..……...…..11

2.4 Table S4: Additive thrombin-inhibitory effects (IC_{50}, μM) of dihydrotanshinone I and cryptotanshinone……...………....……….....…..11

2.5 Table S5: Additive thrombin-inhibitory effects (IC_{50}, μM) of tanshinone IIA and cryptotanshinone..……….....……….……....…...…………………….....…..11

2.6 Table S6: The interactions between TN family and SA family for thrombin inhibitory activity..………....……………………...…………………....…..12

2. Supplementary Figures

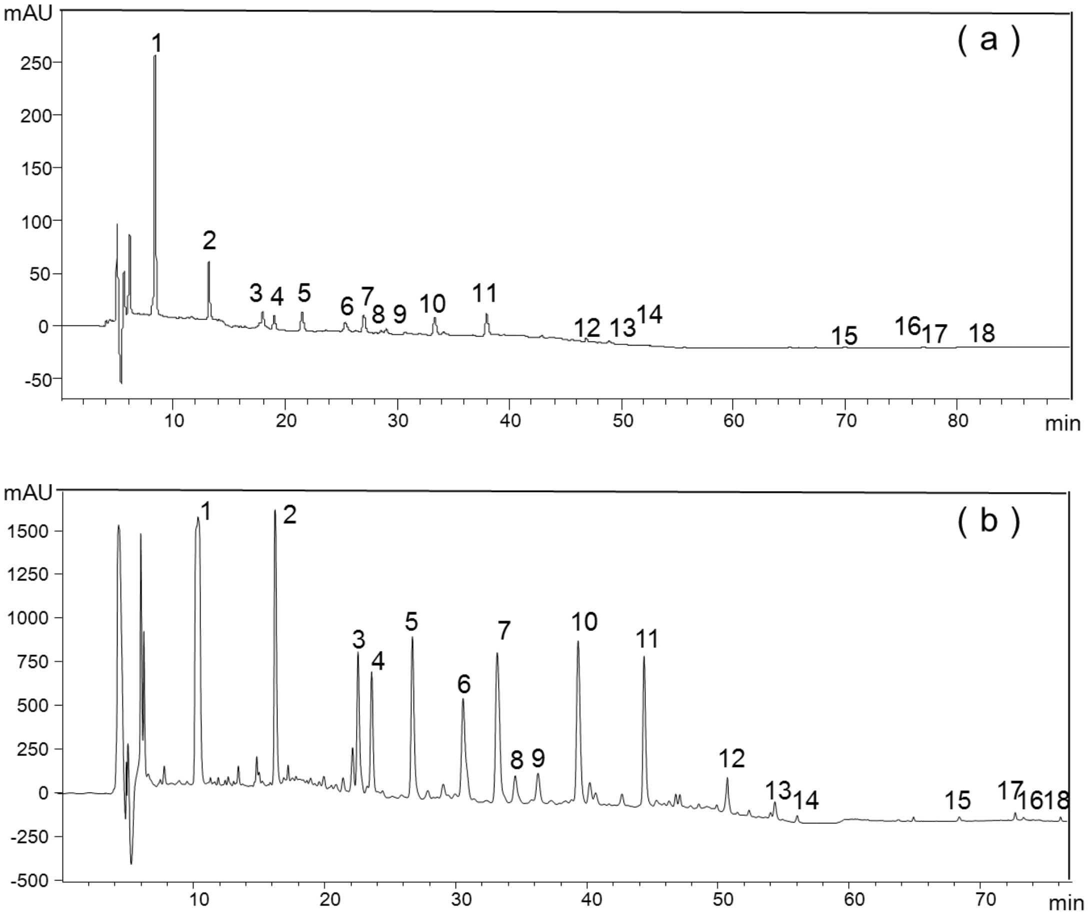

Fig. S1 The chromatographic profiles of DQP performed by analytical column (a) and semi-preparative column (b).

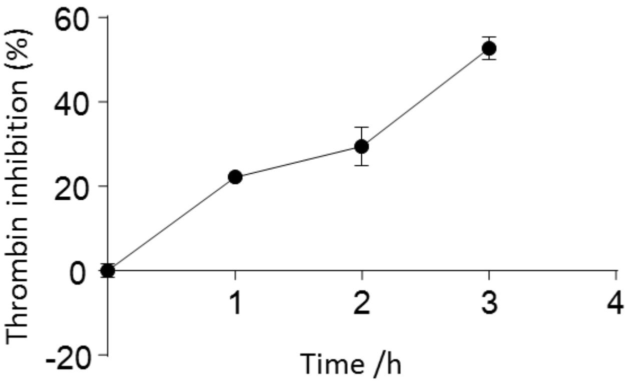

Fig. S2 The thrombin inhibitory activity of salvianolic acid A increased with the time of dissolving in phosphate buffer solution.

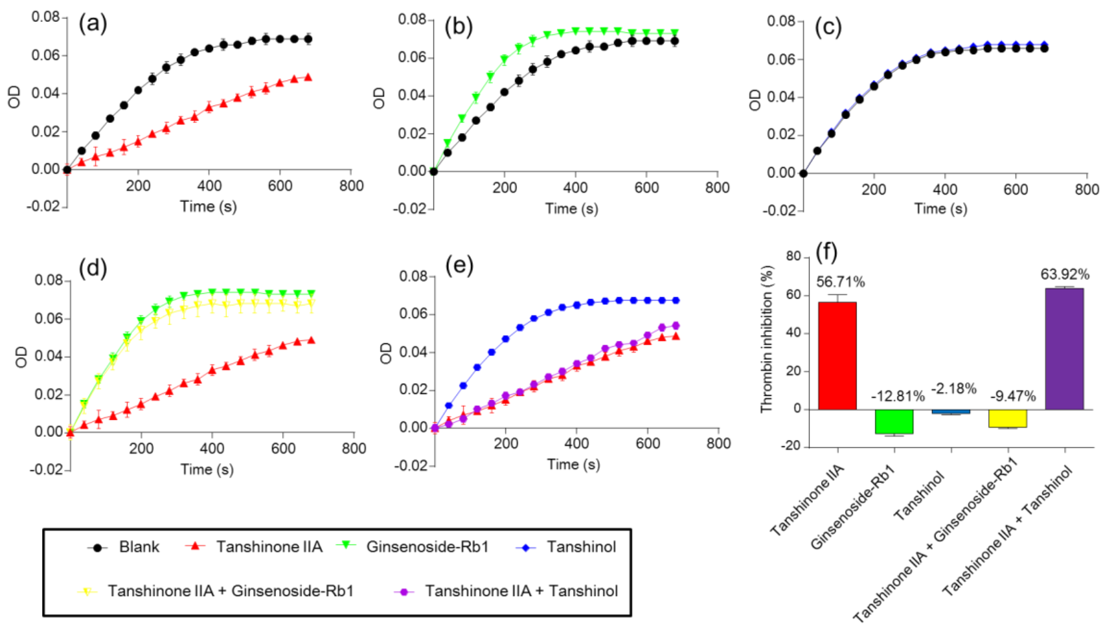

Fig. S3 The kinetics of thrombin catalyzing substrate to product influenced by the representative compounds in different families. (a) The kinetics influenced by tanshinone IIA (TN family, red) and the normal control (black). Tanshinone IIA obviously decreased the reaction rate and delayed the time to reach the plateau. (b) The kinetics influenced by ginsenoside-Rb1 (GS family, green) and the normal control (black). Ginsenoside-Rb1 increased the reaction rate and shortened the time to reach the plateau. (c) The kinetics influenced by tanshinol (SA family, blue) and the normal control (black). Tanshinol had no influence on the kinetics. (d) The kinetics influenced by ginsenoside-Rb1 (green), tanshinone IIA (red), and ginsenoside-Rb1 + tanshinone IIA (yellow). The yellow curve was more similar to the green curve, showing that ginsenoside-Rb1 completely reversed the thrombin inhibition caused by tanshinone IIA. (e) The kinetics influenced by tanshinol (blue), tanshinone IIA (red) and tanshinol + tanshinone IIA (purple). The purple curve was more similar to the red curve, suggesting that tanshinol had no influence on the thrombin inhibition caused by

tanshinone IIA. (f) The quantitative thrombin inhibitory ratios of tanshinone IIA, ginsenoside-Rb1, tanshinol, ginsenoside-Rb1 + tanshinone IIA, and tanshinol + tanshinone IIA.

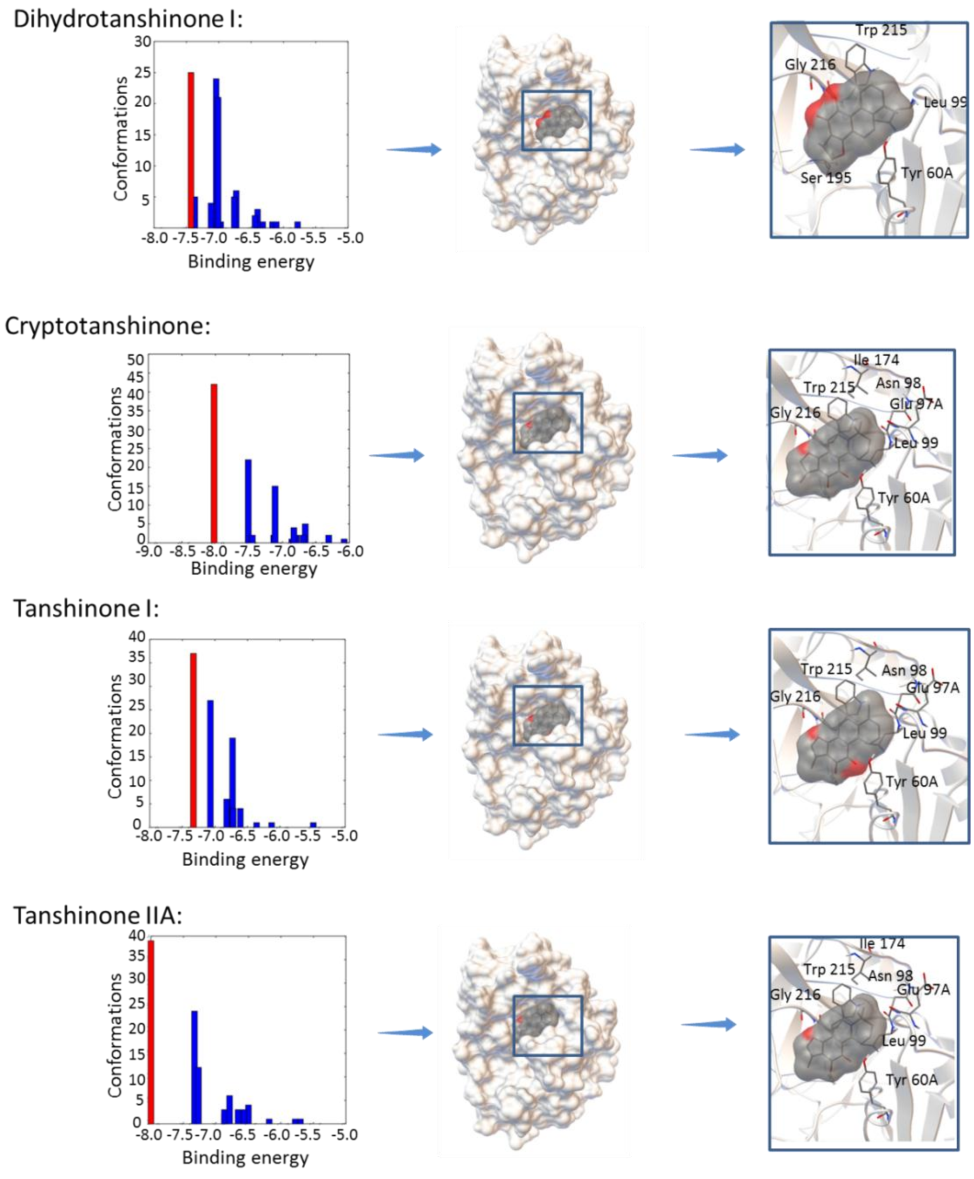

Fig. S4 The docking results of dihydrotanshinone I, cryptotanshinone, tanshinone I and tanshinone IIA to thrombin.

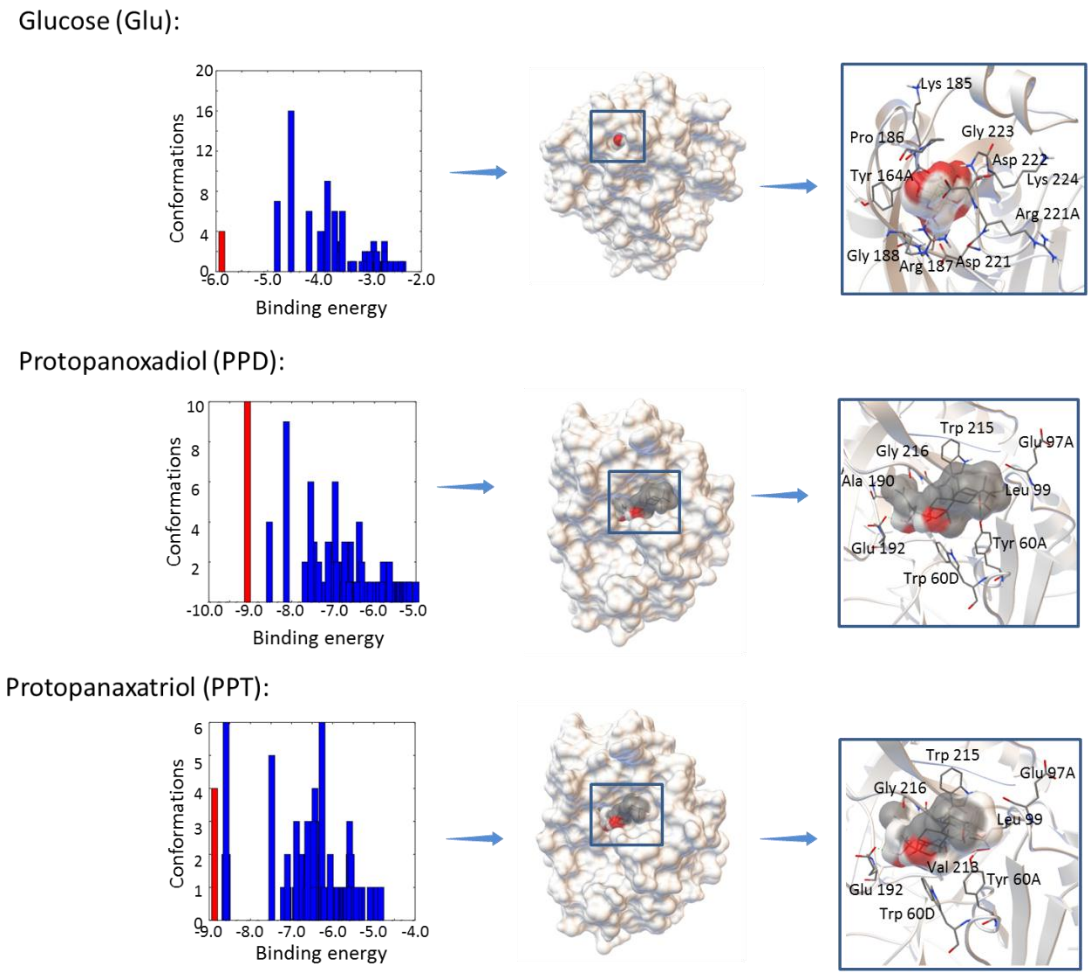

Fig. S5 The docking results of glucose (Glu), protopanaxadiol (PPD) and protopanaxatriol (PPT) to thrombin.

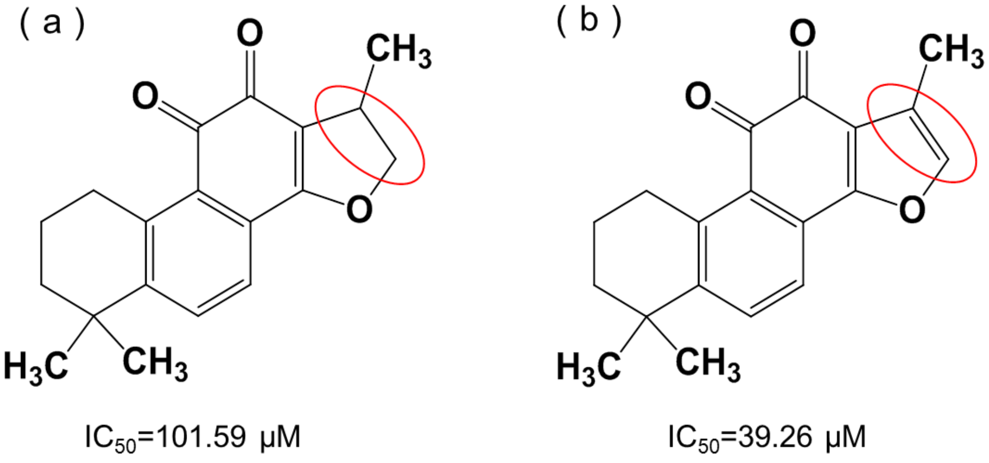

Fig. S6 The structure-activity comparison of cryptotanshinone (a) and tanshinone IIA (b).

3. Supplementary Tables

Table S1 Collection window, collection volume and compound identification of 18 peak fractions.

Fraction No.	Starting Time (min)	Ending Time (min)	Time Window (min)	Collection Volume (mL)	Identification
1	10.21	11.30	1.09	2.18	Tanshinol
2	16.36	17.08	0.72	1.44	Protocatechuic aldehyde
3	22.71	23.43	0.72	1.44	Isolithospermic acid A
4	23.72	24.36	0.64	1.28	Isolithospermic acid B
5	26.73	27.87	1.14	2.28	Salvianolic acid D
6	30.57	31.62	1.05	2.10	Salvianolic acid G
7	33.08	34.43	1.35	2.70	Rosmarinic acid
8	34.43	35.61	1.18	2.36	Lithospermic acid
9	36.31	37.31	1.00	2.00	Ginsenoside-Rg1
10	39.35	40.32	0.97	1.94	Salvianolic acid B
11	44.07	45.43	1.36	2.72	Salvianolic acid A
12	50.63	51.53	0.90	1.80	Ginsenoside-Rb1
13	54.53	55.13	0.60	1.20	Ginsenoside-Rh1
14	56.18	56.94	0.76	1.52	Ginsenoside-Rd
15	68.49	69.01	0.52	1.04	Dihydrotanshinone I
17	72.60	73.38	0.78	1.56	Tanshinone I
16	73.38	74.16	0.78	1.56	Cryptotanshinone
18	76.19	76.79	0.60	1.20	Tanshinone IIA

Table S2 Comparison of the peak areas in DQP extract and the reconstructed library.

Peak No.	t_R	Compound	Chemical family	Peak area	
				Herbal extract	Reconstructed library
1	8.32	Tanshinol	SA	2531	2184
2	13.16	Protocatechuic aldehyde	SA	605	2044
3	18.01	Isolithospermic acid A	SA	281	2593
4	19.00	Isolithospermic acid B	SA	199	2301
5	21.48	Salvianolic acid D	SA	310	2282
6	25.37	Salvianolic acid G	SA	197	2601
7	27.00	Rosmarinic acid	SA	313	2377
8	28.60	Lithospermic acid	SA	48	2408
9	29.06	Ginsenoside-Rg1	GS	80	218
10	33.40	Salvianolic acid B	SA	308	2284
11	37.98	Salvianolic acid A	SA	371	2534
12	46.89	Ginsenoside-Rb1	GS	44	224
13	50.31	Ginsenoside-Rh1	GS	25	284
14	52.36	Ginsenoside-Rd	GS	6	247
15	69.95	Dihydrotanshinone I	TN	8	746
16	76.25	Cryptotanshinone	TN	<5	721
17	77.03	Tanshinone I	TN	9	783
18	82.12	Tanshinone IIA	TN	5	754

Table S3 Additive thrombin-inhibitory effects (IC_{50}, μM) of tanshinone IIA and dihydrotanshinone I.

Tanshinone IIA	Dihydrotanshinone I	C_{IIA}:C_{Dih}	Mixture	CI	Interaction
39.26 ± 0.96	92.46 ± 4.89	1:10	94.77 ± 4.19	1.09	Additive
		1:5	75.68 ± 0.79	1.07	Additive
		1:2	67.70 ± 2.21	1.01	Additive
		1:1	56.82 ± 2.56	1.08	Additive
		2:1	50.07 ± 1.86	1.02	Additive
		5:1	41.55 ± 2.84	1.02	Additive
		10:1	39.17 ± 4.43	1.05	Additive

Table S4 Additive thrombin-inhibitory effects (IC_{50}, μM) of dihydrotanshinone I and cryptotanshinone.

Dihydrotanshinone I	Cryptotanshinone	C_{Dih}:C_{Cry}	Mixture	CI	Interaction
92.46 ± 4.89	101.59 ± 3.84	1:10	100.08 ± 4.35	1.01	Additive
		1:5	99.72 ± 2.65	1.01	Additive
		1:2	93.67 ± 3.41	0.97	Additive
		1:1	88.59 ± 4.72	0.93	Additive
		2:1	92.24 ± 1.55	0.99	Additive
		5:1	91.57 ± 0.96	1.00	Additive
		10:1	84.97 ± 1.16	0.93	Additive

Table S5 Additive thrombin-inhibitory effects (IC_{50}, μM) of tanshinone IIA and cryptotanshinone.

Tanshinone IIA	Cryptotanshinone	C_{IIA}:C_{Cry}	Mixture	CI	Interaction
39.26 ± 0.96	101.59 ± 3.84	1:10	96.04 ± 1.3	0.94	Additive
		1:5	80.76 ± 3.29	0.95	Additive
		1:2	66.94 ± 4.12	1.03	Additive
		1:1	61.35 ± 4.70	1.03	Additive
		2:1	50.47 ± 3.53	1.07	Additive
		5:1	44.77 ± 2.15	0.93	Additive
		10:1	44.19 ± 3.85	1.17	Slight Antagonism

Table S6a The interactions between TN family and SA family for thrombin inhibitory activity.

SA family / TN family	**1.Tanshinol**			**2.Protocatechuic aldehyde**			**3.Rosmarinic acid**		
	Thrombin inhibition (%) at different combination ratios			Thrombin inhibition (%) at different combination ratios			Thrombin inhibition (%) at different combination ratios		
	1/4SA+1TN	1/2SA+1TN	1SA+1TN	1/4SA+1TN	1/2SA+1TN	1SA+1TN	1/4SA+1TN	1/2SA+1TN	1SA+1TN
**Dihydrotanshinone I**	45.20 ±1.78	46.97 ±3.71	45.05 ±1.81	40.93 ±4.63	46.83 ±2.78	42.23 ±1.85	56.57 ±4.15	57.85 ±0.77	47.99 ±3.87
	No dose-dependent interaction			No dose-dependent interaction			No dose-dependent interaction		
**Tanshinone I**	65.81 ±2.19	70.16 ±1.62	57.93 ±12.54	70.79 ±0.73	62.64 ±8.04	66.35 ±2.44	59.43 ±1.77	68.81 ±10.26	60.08 ±3.70
	No dose-dependent interaction			No dose-dependent interaction			No dose-dependent interaction		
**Cryptotanshinone**	42.88 ±3.28	40.36 ±1.66	46.06 ±3.39	36.83 ±2.15	47.76 ±1.20	46.94 ±6.49	58.23 ±3.83	59.74 ±2.61	49.95 ±1.26
	No dose-dependent interaction			No dose-dependent interaction			No dose-dependent interaction		
**Tanshinone IIA**	61.99 ±2.77	56.18 ±2.36	63.92 ±1.16	59.17 ±1.78	67.50 ±1.18	65.00 ±4.71	61.24 ±2.60	62.45 ±0.30	44.92 ±2.96
	No dose-dependent interaction			No dose-dependent interaction			No dose-dependent interaction		

Table S6b The interactions between TN family and SA family for thrombin inhibitory activity (continued).

SA family / TN family	**4.Lithospermic acid**			**5.Salvianolic acid B**			**6.Salvianolic acid A**		
	Thrombin inhibition (%) at different combination ratios			Thrombin inhibition (%) at different combination ratios			Thrombin inhibition (%) at different combination ratios		
	1/4SA+1TN	1/2SA+1TN	1SA+1TN	1/4SA+1TN	1/2SA+1TN	1SA+1TN	1/4SA+1TN	1/2SA+1TN	1SA+1TN
**Dihydrotanshinone I**	56.80 ±0.91	60.57 ±1.81	53.34 ±2.01	43.90 ±3.45	56.10 ±3.45	50.61 ±0.86	46.95 ±2.62	47.32 ±1.58	45.76 ±1.90
	No dose-dependent interaction			No dose-dependent interaction			No dose-dependent interaction		
**Tanshinone I**	71.42 ±9.86	71.78 ±2.93	55.28 ±6.99	75.24 ±1.01	82.93 ±3.05	68.76 ±2.72	70.54 ±1.26	51.61 ±1.26	56.78 ±0.00
	No dose-dependent interaction			No dose-dependent interaction			No dose-dependent interaction		
**Cryptotanshinone**	47.50 ±2.78	63.28 ±2.78	60.94 ±3.91	36.22 ±1.01	43.83 ±3.62	39.58 ±0.82	50.52 ±1.40	47.18 ±1.34	64.26 ±0.46
	No dose-dependent interaction			No dose-dependent interaction			No dose-dependent interaction		
**Tanshinone IIA**	40.46 ±0.89	48.54 ±3.44	45.08 ±2.54	74.99 ±0.87	71.69 ±2.86	76.96 ±0.95	70.58 ±2.28	51.71 ±2.23	62.32 ±2.23
	No dose-dependent interaction			No dose-dependent interaction			No dose-dependent interaction		
